# Impact of opioid law on prescriptions and satisfaction of pediatric burn and orthopedic patients: An epidemiologic study

**DOI:** 10.1371/journal.pone.0294279

**Published:** 2023-11-16

**Authors:** Megan Armstrong, Jonathan I. Groner, Julie Samora, Vanessa A. Olbrecht, Nguyen K. Tram, Dana Noffsinger, Edward W. Boyer, Henry Xiang

**Affiliations:** 1 Center for Pediatric Trauma Research, The Abigail Wexner Research Institute at Nationwide Children’s Hospital, Columbus, OH, United States of America; 2 Center for Injury Research and Policy, The Abigail Wexner Research Institute at Nationwide Children’s Hospital, Columbus, OH, United States of America; 3 Department of Pediatric Surgery, Nationwide Children’s Hospital, Columbus, OH, United States of America; 4 Department of Pediatrics, The Ohio State University College of Medicine, Columbus, OH, United States of America; 5 Department of Orthopedics, Nationwide Children’s Hospital, Columbus, OH, United States of America; 6 Department of Anesthesiology and Pain Medicine, Nationwide Children’s Hospital, Columbus, OH, United States of America; 7 Department of Emergency Medicine, The Ohio State University College of Medicine, Columbus, OH, United States of America; Sheikh Hasina National Institute of Burn & Plastic Surgery, BANGLADESH

## Abstract

**Objectives:**

The objective of this study was to determine the reduction in prescribed opioid pain dosage units to pediatric patients experiencing acute pain and to assess patient satisfaction with pain control 90-day post discharge following the 2017 Ohio opioid prescribing cap law.

**Methods:**

The retrospective chart review included 960 pediatric (age 0–18 years) burn injury and knee arthroscopy patients treated between August 1, 2015-August 31, 2019. Prospectively, legal guardians completed a survey for a convenience sample of 50 patients. Opioid medications (days and morphine milligram equivalents (MMEs)/kg) prescribed at discharge before and after the Ohio law implementation were collected. Guardians reported experience and satisfaction with their child’s opioid prescription at 90-days post discharge.

**Results:**

From pre-law to post-law, there was a significant decrease (p<0.001) within the burn and knee cohorts in the median days (1.7 to 1.0 and 5.0 to 3.8, respectively) and median total MMEs prescribed (15.0 to 2.5 and 150.0 to 90.0, respectively). An interrupted time series analysis revealed a statistically significant decrease in MMEs/kg and days prescribed at discharge when the 2017 Ohio opioid prescription law went into effect, with an abrupt level change. Prospectively, more than half of participants were satisfied (72% burn and 68% knee) with their pain control and felt they received the right amount of medication (84% burn and 56% knee). Inpatient opioid use was not changed pre- and post-law.

**Conclusions:**

Discharge opioids prescribed for pediatric burn and knee arthroscopy procedures has decreased from 2015–2019. Caregivers varied greatly in their satisfaction with pain control and the amount of opioid prescribed.

## Introduction

Pediatric exposure to prescribed opioid analgesics is associated with striking rates of opioid abuse that may be as great as 33% among adolescents and young adults [1]. Nearly half of opioid prescriptions written to pediatric patients are considered high risk [2]. In addition to the potential for opioid misuse, overdose, and death, opioids prescribed to children carry the additional risk of misuse by relatives [2]. Even among several states with dramatic rates of opioid abuse and overdose death, Ohio had the second highest rate of opioid overdose death in 2017 [3]. The United States Department of Health and Human Services declared the opioid crisis a public health emergency in 2017 [4–6]. The 2020 national data reported death rates for any opioid overdose as 20.8 per 100,000 US population, compared with an all motor vehicle death rate of 12.4 and all firearms death rate of 13.7 [7]. Prescribed opioids played an important role in opioid overdoses [8]. The age-adjusted rate of deaths from synthetic opioids other than methadone increased by 1,040% from 2013 to 2019 [9]. Opioid drug overdose prevention and research were among the top priorities for local, state, and federal agencies [10, 11] until the COVID-19 pandemic shifted priorities. Multiple strategies have been implemented to fight the US opioid crisis [12–14]. As of 2021, national systematic legal research found that 38 states had opioid prescribing laws, 11 had pill mill laws, 33 had enacted mandatory prescription drug monitoring program (PDMP) query laws, and 22 had compulsory PDMP enrollment laws [13, 14]. By presuming that exposure is a leading risk factor for adverse events, opioid dose restrictions would reduce outcomes of addiction and overdose and improve the safety of this effect class of analgesics [12, 13]. The Ohio opioid cap law, enacted on August 31, 2017 [15], limited the total morphine milligram equivalents (MME) to an average of 30 MME/day and no more than five days of prescribed opioids for minors [15]. The anticipated benefit of cap laws in preventing opioid overdose, death, and addiction was counterbalanced by fears that decreased opioid prescribing could hurt patient satisfaction.

Assessing the impact of state prescribing cap laws on opioids prescriptions is essential, given the large amount of opioids prescribed for postoperative pain management and the risk for addiction or overdose. Equally significant is the lack of studies on patients’ opioid utilization, pain management satisfaction, and opinions regarding pain medications after hospital discharge. Maintaining a delicate balance between reducing opioids while also ensuring effective pain management is critical, since poorly managed acute postsurgical pain has the risk to transition to chronic pain [16, 17], which could cause serious psychological problems in children with impacts reaching well into adulthood [18, 19]. In this study, we aimed to assess the impact of the Ohio opioid prescribing cap law on opioid utilization during the hospital stay, opioid and pain medications prescribed at discharge, and patient pain medication experience 90 days after hospital discharge. We hypothesized: 1) pediatric doctors prescribed significantly fewer opioids at discharge after the Ohio opioid prescribing cap law; 2) the majority of patients (≥ 75% of surveyed patients) were satisfied with their pain control after discharge.

## Methods

This study compared the amount of opioid analgesics prescribed to children following enactment of the Ohio Opioid Cap Law with historical controls. We conducted this study at a Nationwide Children’s Hospital, a referral tertiary care children’s hospital in central Ohio. We selected two patient cohorts commonly prescribed opioid pain medications: burn injury and knee arthroscopy patients. Our inclusion criteria were children aged 0–18 years of age who received a prescription for opioid analgesics following a) burn care in inpatient or outpatient settings or b) knee arthroscopy. We excluded patients who were aged 19 years or older, received no opioid analgesics (either inpatient or as a discharge prescription), underwent knee arthroscopy with simultaneous anterior cruciate ligament reconstruction, were admitted for more than 24 hours following knee arthroscopy, were minor in foster care (Phase 2), or were unable to communicate in English (Phase 2). The knee arthroscopy exclusion criteria were required in order to ensure a more homogenous and standardized knee cohort. The Nationwide Children’s Hospital institutional review board approved this study in August 2020. Informed consent was waived for Phase 1 and verbal consent was obtained for Phase 2. This work is reported as per STROBE guidelines and a STROBE checklist can be found in **S1 Appendix**.

### Phase 1 –Retrospective chart review

We compared the pre-law (August 1, 2015, to August 31, 2017) and post-law periods (September 1, 2017, to August 31, 2019). ICD-9 codes 940–949 or ICD-10 code T30.0 identified burn patients, and the Current Procedural Terminology code 29873 identified knee arthroscopy procedures. Burn cases were filtered for the patient’s initial visit for the injury and randomly selected using a simple random sample (n = 150 pre-law and n = 150 post-law). All knee cases which met the inclusion criteria were included.

The chart review collected demographic/injury variables (i.e., injury type, such as knee injury or burn); admission date/time; discharge date/time; date of birth; sex; race; ethnicity; weight in kilograms; location of burn treatment (i.e., ED, admitted, outpatient burn clinic); percent total body surface area (%TBSA) burned; burn depth; discharge pain medication (i.e., opioid prescribed (name, dose, quantity), acetaminophen (yes/no), acetaminophen coformulated with an opioid analgesic, ibuprofen, other nonsteroidal anti-inflammatory medication) and opioid medications administered during injury care; and opioid medications administered during treatment (name, frequency, quantity, dose) per 24 hours (up to 120 hours)]. Using the Centers for Disease Control and Prevention (CDC) opioid conversion factors [20], MMEs/kg were calculated per 24 hours, as well as total administered MMEs, total discharge MMEs prescribed, days prescribed, and MMEs/day.

### Phase 2 –Prospective survey

Telephone surveys were conducted during the post law era, between October 2020 to December 2021. Beginning with discharges occurring in July 2020, a telephone survey was conducted 90 days after hospital discharge using Glaser’s et al. approach [21]. For prospective data collection, we used the identical protocol inclusion criteria, exclusion criteria, and ICD search terms to identify study participants. Hospital discharge lists identified burn and knee arthroscopy patients discharged that month and eligible for contact. The survey was conducted among a convenience sample of adult parents/caregivers of 50 pediatric patients (n = 25 burn patients and n = 25 knee arthroscopy patients) to assess quantity of opioids utilized, duration of opioid use, number of refills, patients’ experiences with refills, and caregivers’ perceptions of pain control after discharge. Caregivers were questioned about nonprescription and alternative pain control and the disposal of any remaining opioid medications. Utilization was assessed by asking caregivers to count or estimate the number of remaining pills or the remaining liquid volume. A Research Electronic Data Capture (REDCap) recorded all survey responses [22, 23].

Eligible patients received an invitation letter with an opt-out option for families who did not want to participate. We contacted all families who did not opt out by telephone 90 days post-discharge to introduce the survey and obtain verbal consent. Anyone who did not answer the phone received a voicemail with a brief study explanation and a callback number. Three contact attempts were made for each eligible child before excluding them from the study. The comparison analysis of eligible patients who declined to participate, were never reached via phone, and recruited showed that these groups did not differ significantly in terms of demographics, length of hospital stay, and prescribed hydrocodone vs oxycodone (**S1 Table**). After receiving 25 completed surveys and information saturation was reached for a cohort, that cohort was no longer eligible for sampling. To minimize patient/family and prescriber bias [24], neither surgeons nor patients were informed of the upcoming telephone survey during their hospital stay, nor did they know which patients would be selected.

### Study outcomes and confounding variables

#### Primary outcome

Our primary outcomes were MME/kg and the number of days of opioids prescribed at discharge pre- and post-cap law.

#### Secondary outcomes and confounding variables

To assess the spillover effect of the opioid cap law, we also evaluated the opioid pain medication administered during both the child’s outpatient and inpatient hospital stay. We calculated MMEs/kg per 24-hours to control for patient’s body weight, using multiple pain medications and varying treatment durations. Confounding variables included sex, age, race/ethnicity, medical conditions (burn or knee injury), %TBSA for burn patients, and days to enacting the 2017 Ohio opioid prescribing cap law.

#### Statistical analysis

We compared patient demographics (sex, age, race/ethnicity) by medical conditions (burn or knee injury) and %TBSA for burn patients pre- and post-prescription cap law. We identified non-parametric data as median and interquartile ranges (IQR). Categorical variables were reported as frequencies and percentages (%). Where appropriate, a two-sample t-test or the Wilcoxon rank-sum test investigated the differences in continuous variables, and Chi-square test evaluated the differences for categorical variables.

Interrupted time series analysis estimated the effect of the cap law on the amount and duration of opioid prescribed at discharge while controlling for sex, age, race/ethnicity, medical conditions (burn or knee injury), and days to enacting the cap law. A p-value <0.05 from statistical tests was considered statistically significant. All statistical analyses were performed using Prism v9 for Windows (GraphPad Software, San Diego, CA, USA) or Python v3.9 (Python Software Foundation, Wilmington, DE).

## Results

**Fig 1:**
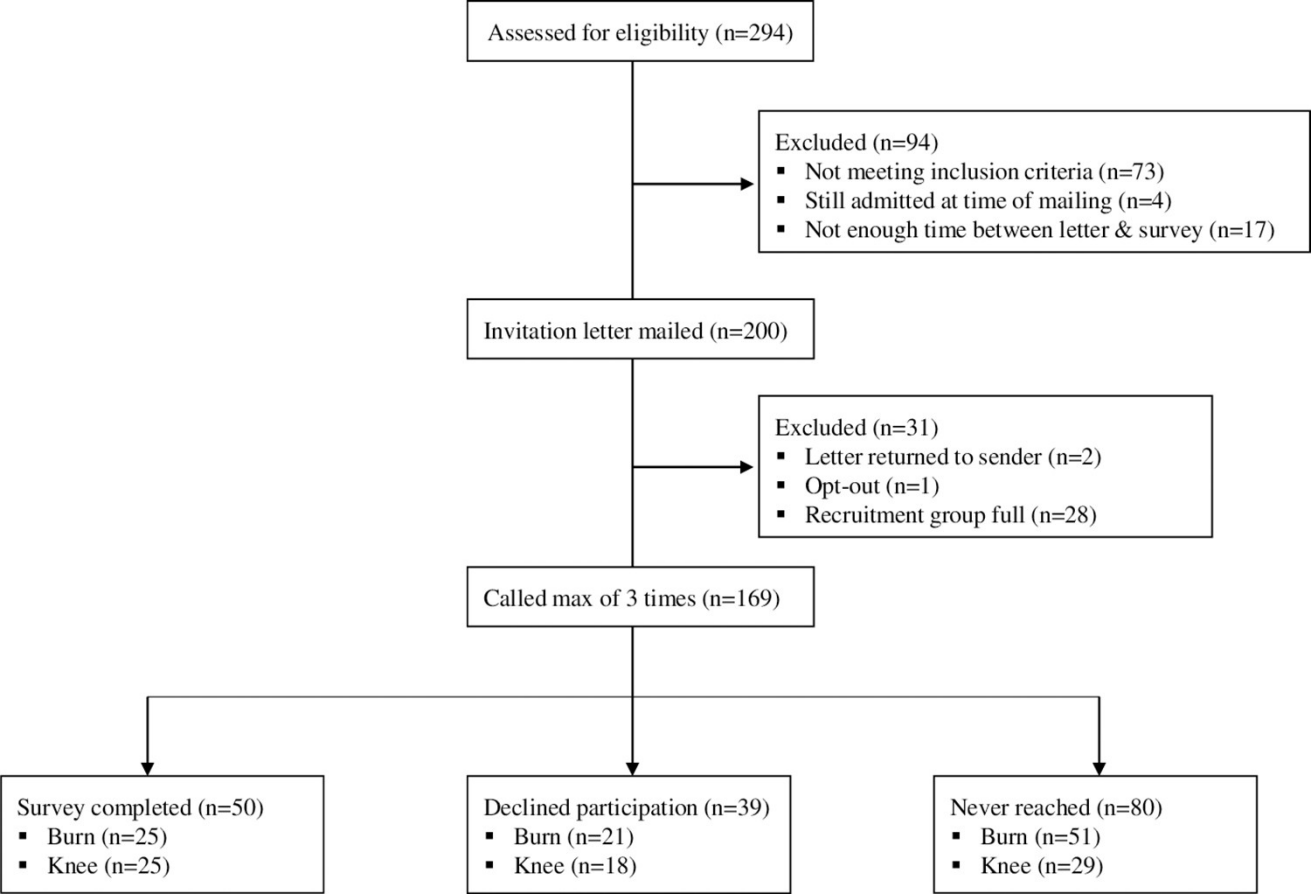
CONSORT diagram for prospective survey recruitment.

Of the 200 families deemed eligible and having a letter mailed to them (**Fig 1**), 169 patients received at least one call. Fifty recruited participants completed the survey (n = 25 burn injury and n = 25 knee arthroscopy), 39 declined to participate, and 80 were unreachable.

Pre-law (n = 150) and post-law (n = 150) burn patient cohorts were comparable in sex, age, race/ethnicity, %TBSA burned, and the total MME/kg administered per 24 hours of hospitalization (**Table 1**). Over half (61%, n = 182) of burn patients received an opioid prescription at discharge (there was no statistically significant difference in the number of patients who did not receive a prescription pre- and post-law). Median days prescribed at discharge significantly decreased from 1.7 to 1.0 days (p<0.001), and median total MMEs prescribed decreased from 15.0 to 2.5 MME (p<0.001) pre- and post-law, respectively. Discharge prescriptions exceeding 30 MME/day did not significantly change after the cap law, as most prescriptions pre-law (91.2%) and post-law (95.0%) were less than the cap. For further confirmation of results, discharge prescriptions were stratified by age (<11 years and 11+ years) and can be reviewed in **S2 Table**.

**Table 1:** Patient characteristics by medical conditions pre-and post-prescription law.

Characteristics	Overall	Pre-law	Post-law	P-value**	
	N(%)	N(%)	N(%)		
**BURN INJURY**	300	150	150		
**Sex**				
Male	174 (58.0)	83 (55.3)	91 (60.7)	0.41	
Female	126 (42.0)	67 (44.7)	59 (39.3)		
**Age (Years)**				
<2	102 (34.0)	56 (37.3)	46 (30.7)	0.62	
2–5	93 (31.0)	46 (30.7)	47 (31.3)		
6–11	65 (21.7)	30 (20.0)	35 (23.3)		
12–17	40 (13.3)	18 (12.0)	22 (14.7)		
**Race/ethnicity**				
White, non-Hispanic	168 (56.0)	82 (54.7)	86 (57.3)	0.66	
Black, non-Hispanic	85 (28.3)	41 (27.3)	44 (29.3)		
Hispanic	14 (4.7)	9 (6.0)	5 (3.3)		
Other	33 (11.0)	18 (12.0)	15 (10.0)		
%TBSA burned*				
TBSA<5%	220 (81.2)	101 (80.2)	119 (82.1)	0.92	
TBSA 5–9%	34 (12.5)	17 (13.5)	17 (11.7)		
TBSA 10–19%	14 (5.2)	7 (5.6)	7 (4.8)		
TBSA ≥20%	3 (1.1)	1 (0.8)	2 (1.4)		
**Total MMEs per kg of body weight taken in 24 h of hospitalization**				
Median (IQR)	0.5 [0.2, 4.0]	0.9 [0.2, 4.1]	0.4 [0.2, 3.9]	0.61	
**DISCHARGE PRESCRIPTIONS (n = 182)**				
**No. of days opioid prescribed at discharge**				
Median (IQR)	1.6 [0.0, 5.0]	1.7 [0.0, 10.0]	1.0 [0.0, 2.5]	**<0.001**	
**Total MMEs prescribed at discharge**				
Median (IQR)	7.5 [0.0, 22.5]	15.0 [0.0, 30.0]	2.5 [0.0, 15.0]	**<0.001**	
**Discharge prescription exceeding 30 MME per day**				
No (%)	169 (92.9)	93 (91.2)	76 (95.0)	0.48	
Yes (%)	13 (7.1)	9 (8.8)	4 (5.0)		
**Discharge acetaminophen prescribed**				
No (%)	18 (10.5)	12 (12.2)	6 (8.1)	0.53	
Yes (%)	154 (89.5)	86 (87.8)	68 (91.9)		
**Discharge acetaminophen combo medication prescribed**				
No (%)	150 (87.2)	82 (83.7)	68 (91.9)	0.17	
Yes (%)	22 (12.8)	16 (16.3)	6 (8.1)		
**Discharge ibuprofen prescribed**				
No (%)	161 (93.6)	93 (94.9)	68 (91.9)	0.53	
Yes (%)	11 (6.4)	5 (5.1)	6 (8.1)		
**KNEE INJURY**	630	204	426		
**Sex**				
Male	310 (49.2)	86 (42.2)	224 (52.6)	**0.02**	
Female	320 (50.8)	118 (57.8)	202 (47.4)		
**Age (Years)**				
<2	1 (0.2)	-	1 (0.2)	0.91	
2–5	3 (0.5)	1 (0.5)	2 (0.5)		
6–11	83 (13.2)	26 (12.7)	57 (13.4)		
12–17	543 (86.2)	177 (86.8)	366 (85.9)		
**Race/ethnicity**				
White, non-Hispanic	464 (73.7)	155 (76.0)	309 (72.5)	0.08	
Black, non-Hispanic	100 (15.9)	36 (17.6)	64 (15.0)		
Hispanic	25 (4.0)	3 (1.5)	22 (5.2)		
Other	41 (6.5)	10 (4.9)	31 (7.3)		
**Total MMEs per kg of body weight taken in 24 h of hospitalization**				
Median (IQR)	3.4 [2.3, 4.2]	3.5 [2.5, 4.2]	3.4 [2.2, 4.2]	0.24	
**DISCHARGE PRESCRIPTIONS (n = 611)**				
**No. of days prescribed at discharge**				
Median (IQR)	5.0 [2.5, 5.0]	5.0 [5.0, 10.0]	3.8 [1.6, 5.0]	**<0.001**	
**Total MMEs prescribed at discharge**				
Median (IQR)	100.0 [50.0, 150.0]	150.0 [100.0, 200.0]	90.0 [45.0, 100.0]	**<0.001**	
**Discharge prescription exceeding 30 MME per day**				
No (%)	590 (96.6)	188 (95.4)	402 (97.1)	0.41	
Yes (%)	21 (3.4)	9 (4.6)	12 (2.9)		
**Discharge acetaminophen prescribed**				
No (%)	448 (79.0)	154 (79.4)	294 (78.8)	0.96	
Yes (%)	119 (21.0)	40 (20.6)	79 (21.2)		
**Discharge acetaminophen combo medication prescribed**				
No (%)	26 (4.6)	4 (2.1)	22 (5.9)	0.06	
Yes (%)	541 (95.4)	190 (97.9)	351 (94.1)		
**Discharge ibuprofen prescribed**				
No (%)	430 (75.8)	139 (71.6)	291 (78.0)	0.12	
Yes (%)	137 (24.2)	55 (28.4)	82 (22.0)		

^a^ p<0.05 considered significant (bold)

^b^ 29 subjects missing TBSA

SD = Standard Deviation; IQR = interquartile range; %TBSA: % total body surface area; MME: morphine milligram equivalents

The knee cohort (n = 204 pre-law and n = 426 post-law) was comparable in age, race/ethnicity, and the total MME/kg administered per 24 hours of hospitalization (**Table 1**); however, the sex distribution was significantly shifted toward males post-law (p = 0.02). Most (n = 611) of the knee cohort received an opioid prescription at discharge (there was no statistically significant difference in the number of patients who did not receive a prescription pre- and post-law). Median days prescribed at discharge significantly decreased from 5.0 to 3.8 days (p<0.001), and median total MMEs prescribed decreased from 150.0 to 90.0 MME (p<0.001) pre- and post-law, respectively. Discharge prescriptions exceeding 30 MME/day did not significantly change with the cap law, as most prescriptions pre-law (95.4%) and post-law (97.1%) were less than the cap.

**Fig 2:**
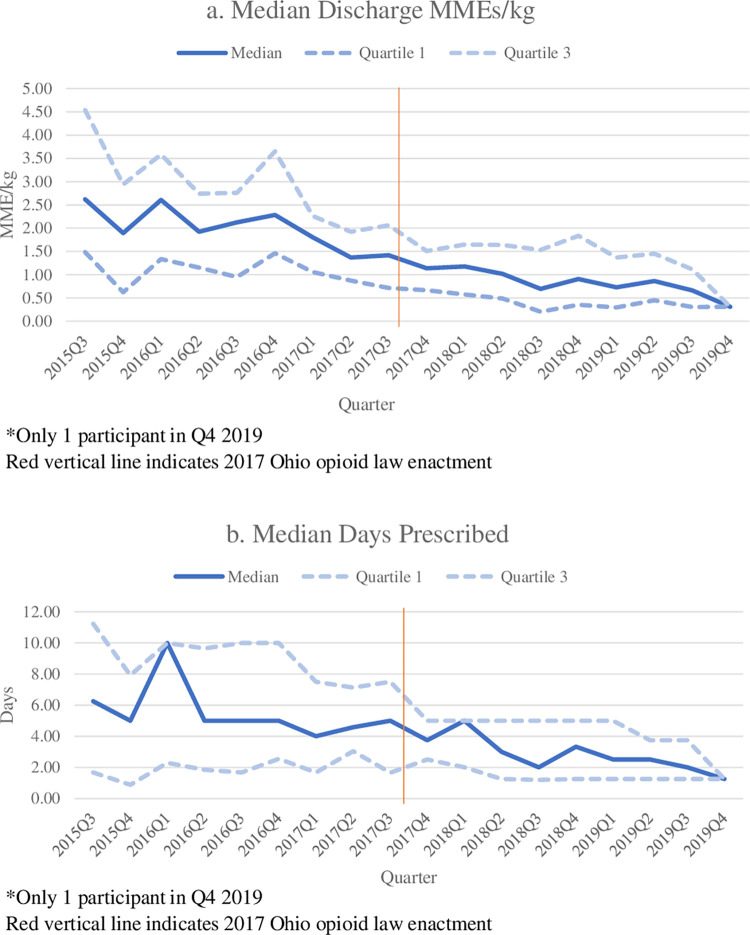
**A & B**: Median MME/kg and days prescribed at discharge for combined burn injury and knee arthroscopy, pre- and post-law.

**Fig 3:**
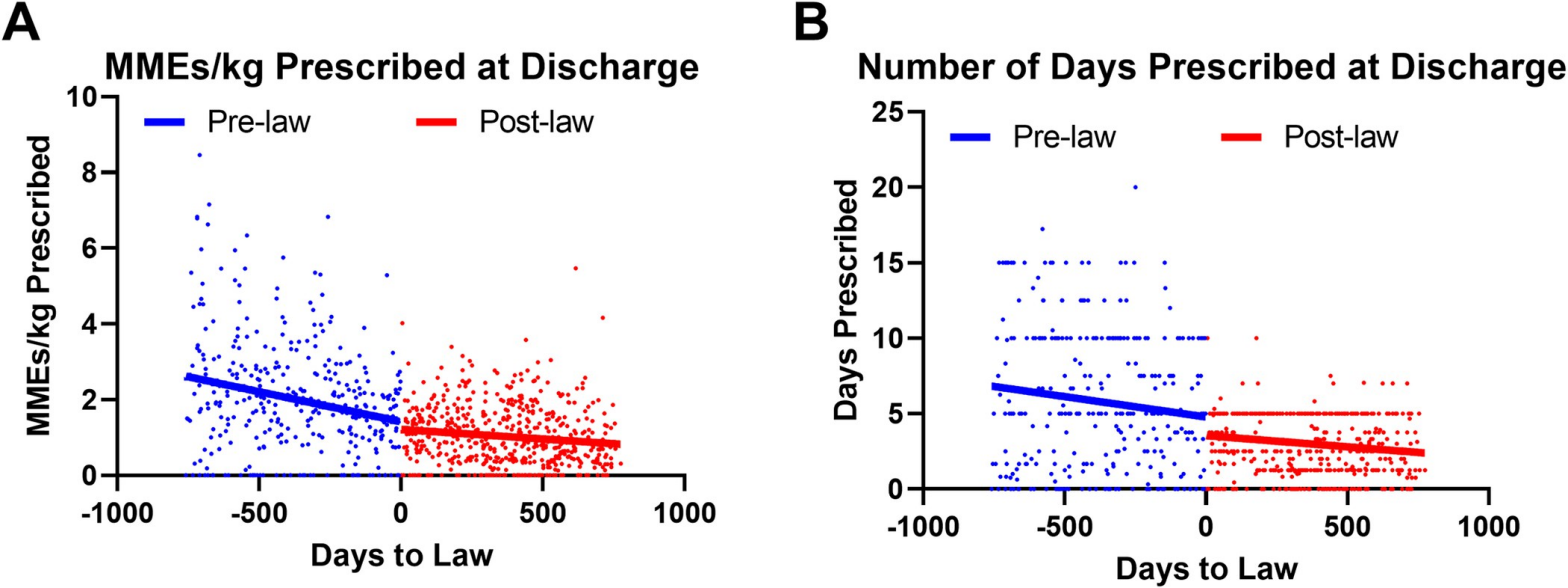
Number of days and MMEs/kg prescribed at discharge by days to law change.

In the earliest quarter of our study, Q3 2015, patients were prescribed a median 2.62 MME/kg [quartile 1 = 1.49 and quartile 3 = 4.53] and 6.25 days (quartile 1 = 1.67 and quartile 3 = 11.24) at discharge (**Fig 2A & 2B**). In Q3 2017 when the cap law was enacted, patients were prescribed a median 1.42 MME/kg (quartile 1 = 0.17 and quartile 3 = 2.06) and 5.00 days (quartile 1 = 1.65 and quartile 3 = 7.50) of opioids. We found that variability in opioid prescribing practices decreased toward the end of our study period. Interrupted time series analyses confirmed that the amount and duration of opioids prescribed at discharge decreased steadily during both pre- and post-law periods (**Fig 3**). MMEs/kg and the number of prescribed days exhibited an abrupt downward change when the law went into effect (days to law = 0), with a significantly reduced post-law slope for MMEs/kg prescribed.

Burn patients used prescribed opioid analgesic medications for a median 6.5 days (min = 0, max = 14), while the knee patients used their opioid analgesics for a median 2 days (min = 0, max = 7) after discharge (**Table 2**). A substantial proportion of participants (e.g., 72% of burn patients; 68% of arthroscopy patients) were very satisfied with their pain control after discharge. Eighty-four percent of burn patients and 56% of knee patients felt they received the right dosage of pain medication. Interestingly, 12% of burn patients and 32% of knee patients reported being prescribed too much medication. In comparison, 4% of burn patients and 8% of knee patients felt their prescribed pain medications were insufficient. Among the knee cohort, N = 3 patients did not fill their prescription, represented in the “none leftover” group, leaving 68% of burn patients and 52% of knee patients reported having leftover medication. Families reported keeping the medication (24% burn and 12% of knee), giving it to a drug takeback event (12% burn and 16% knee), dissolving the medication with a pharmacy-provided kit (12% burn and 12% knee), and throwing it in the trash or flushing it (16% burn and 12% knee).

**Table 2:** Opioid medication use and patient satisfaction after hospital discharge.

	Burn Injury (n = 25)		Knee Arthroscopy (n = 25)		
Characteristic	No.	%	No.	%	
**Sex**					
Male	12	48%	13	52%	
Female	13	52%	12	48%	
**Race**					
White	16	64%	21	84%	
Black	4	16%	3	12%	
Other	5	20%	1	4%	
**Age (y)**, Median (Min, Max)	2	(0,15)	15	(10,17)	
**How many days after discharge did your child STOP taking their prescription pain medications?**					
Median (Min, Max)	6.5	(0,14)	2	(0,7)	
**Did your child receive any prescription pain medications AFTER leaving the hospital? (i.e., refills or new medications)**				
Yes	4	16%	0	0%	
No	21	84%	25	100%	
**On a scale of 0–10, did this medication adequately control your child’s pain after discharge? 0 = not controlled; 10 = completely controlled**				
Median (Min, Max)	8.5	(5,10)	10	(3,10)	
**How satisfied or dissatisfied was your child with their pain control after discharge?**				
Very satisfied	18	72%	17	68%	
Somewhat satisfied	6	24%	5	20%	
Neutral	1	4%	0	0%	
Somewhat dissatisfied	0	0%	3	12%	
**Which statement best describes your child’s experience regarding the pain medication they were prescribed at discharge?**					
Not enough	1	4%	2	8%	
Right amount	21	84%	14	56%	
Too much	3	12%	8	32%	
**What did you do with your child’s leftover prescription opioid medication?**				
None leftover	8	32%	12	48%	
Kept	6	24%	3	12%	
Drug takeback	3	12%	4	16%	
Dissolved with provided kit	3	12%	3	12%	
Threw in trash or flushed	4	16%	3	12%	
Other	1	4%	0	0%	

## Discussion

Legislation to limit opioid use significantly decreased opioid exposure to these vulnerable populations of children and adolescents. After enacting the opioid prescribing cap law, the median number of days and total MMEs of opioids prescribed to pediatric burn and knee injury patients significantly decreased. However, median total MMEs/kg taken within 24 hours of hospitalization did not change significantly; nor did the discharge opioid prescriptions exceeding 30 MME/day, as more than 95% of the prescriptions pre- and post-law were less than 30 MME/day. Variability was found in opioid medication use, pain control satisfaction, the amount of opioids prescribed at discharge, and how leftover opioid medications were handled.

Our study found the median number of days and median total MMEs of opioids prescribed to pediatric burn and knee injury patients significantly decreased after the 2017 Ohio opioid prescribing cap law but that the majority of opioids prescribed at discharge pre-law were already under the new prescribing cap. These findings are consistent with previous single-state adult studies that reported a significant reduction in the volume of opioids dispensed at postoperative discharge [25–28]. Both our study and the previous research by Richards et al. [29] found a consistent declining trend of opioid medications prescribed to pediatric burn patients over several years before the law, suggesting an overall downward trend of opioid prescribing behaviors regardless of the institution of new legislature. Thus, it is challenging to pinpoint and claim the opioid prescribing cap law was the sole driving force that significantly reduced the prescribed opioid medications. Some investigators have suggested other interventions (e.g., education, hospital-wide and department specific quality improvement projects, hospital and CDC clinical practice guidelines [30] for prescribing opioids) and other opioid prescription laws (pain pill mill laws, PDMP query laws, and PDMP enrollment laws [13, 14]) likely all played important roles in reducing the prescribed opioids among patients [31]. The downward shift at the implementation of the 2017 Ohio opioid prescribing cap law sped up the decreases in prescribing seen prior to the law and suggests a burden of overprescribing prior to the law and less opportunity for abuse and misuse in this population following the implementation.

We found that Ohio’s cap law had no effect on inpatient opioid prescribing. The reasons for this are unclear. Consistency in opioid dosing may be due to the acute need for analgesia early in a course of treatment. Alternatively, ordering of medications may represent ingrained patterns of behavior among inpatient clinical staff, or reflect the lack of change in dosing recommendations for opioid analgesics. Finally, while physicians write orders for opioid administration, the responsibility for inpatient analgesic administration lies in the hands of nursing staff. Placing decisions regarding administration of pain medication in the hands of nurses may minimize the effect of cap laws on opioid utilization among inpatients.

Despite observed decreases in the number of opioids dispensed, a substantial proportion of respondents indicated that physicians nonetheless prescribed too many opioids. Our findings corroborated the study by Wendel et al. that reported more than half of opioids prescribed to pediatric patients were unused after ambulatory knee surgery, even with prescribing guidelines in place [32]. This finding is consistent with previous data that only brief periods of opioid analgesics are needed to control acute, severe pain, even for conditions that require surgical management [33, 34]. Pain is increasingly recognized as a complex biopsychosocial process which pharmacologic treatments alone may be insufficient. The variability in opioid pain medication use, pain control satisfaction, and opinions of parents or guardians about the amount of opioids prescribed at discharge in our study highlight the subjective nature of the pain experience. Neuroscience and biobehavioral studies indicate that pain is a multidimensional experience with sensory-discriminative, affective-motivational, and cognitive-evaluative components [35, 36] for which multimodal non-opioid drugs and nonpharmaceutical pain management approaches are needed [35, 37, 38]. Evidence continues to grow that laws fail to consider prescriber intent, patient-specific factors, and patient/family preference [13, 39], which could lead to unintended consequences of not controlling some patients’ pain well [40] or prescribing more medication than needed. The underlying biological, cognitive, and behavioral mechanisms of pain, heterogeneous preferences of both prescribers and patients, and adverse consequences at different thresholds for long-term use warrant a precision prescription approach of opioids for pain management. The precision prescription research, which aligns well with the US Precision Medicine Initiative [41], should be funded as a bold national initiative like the recent NIH-funded precision nutrition project [42].

The findings of this study should be interpreted in the context of several limitations. First, this study used data from a single large pediatric medical center in Ohio, potentially limiting the generalizability of findings to other hospitals and medical facilities where pediatric trauma patients were treated or other states that implemented different opioid prescribing cap laws. While our results are consistent with previous single-state studies, large national studies that used medical claims data reported no reduction in prescribed opioids after implementing opioid cap laws. One plausible explanation for the null association of the national studies might be the large variety of heterogeneous medical conditions that masked the significant reduction of prescribed opioids for some subcategories of medical conditions. Thus, no opioid prescribing cap law or opioid prescribing guidelines exist that would be one-size-fits-all.

Second, we clearly defined the periods of pre- and post-Ohio opioid prescribing cap laws; however other opioid limitation laws were implemented in Ohio and across the United States, which could potentially affect the opioid prescribing behaviors of prescribers. Our hospital also implemented quality improvement initiatives that could have contributed to the observed results.

Third, we felt the responses for the prospective surveys had reached a saturation point when our funding ended, however, our sample size is relatively small and results need to be interpreted within the context of the sample size. We were not able to perform significance tests due to the small sample, which could point to points of emphasis for future prescribing efforts to consider. Future studies looking at 90-day opioid medication usage should consider the challenge of obtaining a substantial sample when using these sampling methods.

## Conclusions

The amount and days of opioids prescribed at discharge after the 2017 Ohio prescription opioid cap law were reduced significantly. Inpatient opioid use, however, was not changed. Parents and guardians had varying levels of satisfaction with pain control following discharge and remaining amounts of opioids. More research is needed into personalized precision prescribing to optimize patient pain management.

## Supporting information

S1 Appendix STROBE statement—checklist of items that should be included in reports of cross-sectional studies. (DOCX)

S1 Table Comparison of eligible prospective subjects by those who declined survey, were never reached via phone, and recruited. (DOCX)

S2 Table Discharge opioids prescribed pre- and post-law among patients age <11 years and 11+ years. (DOCX)
