# Supplementary material for: Impact of opioid law on prescriptions and satisfaction of pediatric burn and orthopedic patients: An epidemiologic study
Source: PLoS One. 2023 Nov 16;18(11):e0294279. doi: 10.1371/journal.pone.0294279 (PMC10653505; doi:10.1371/journal.pone.0294279)
Supplement: S1 Table — (DOCX) [file pone.0294279.s002.docx]

**S1 Table**: Comparison of Eligible Prospective Subjects by Those who Declined Survey, Were Never Reached via Phone, and Recruited

| **Characteristics** | **Overall**  **N(%)** | **Declined Survey N(%)** | **Never Reached N(%)** | **Recruited**  **N(%)** | **P-value*** |
| --- | --- | --- | --- | --- | --- |
| **Burn Injury** | 97 | 21 | 51 | 25 |  |
| **Sex** | | | | | |
| Male | 58 (59.8) | 16 (76.2) | 29 (56.9) | 13 (52.0) |  |
| Female | 39 (40.2) | 5 (23.8) | 22 (43.1) | 12 (48.0) | 0.206 |
| **Age**, median [Q1, Q3] | 2.0 [1.0, 6.0] | 2.0 [1.0, 4.0] | 3.0 [1.0, 9.0] | 2.0 [1.0, 6.0] | 0.606 |
| **Race** | | | | | |
| White | 62 (63.9) | 11 (52.4) | 35 (68.6) | 16 (64.0) |  |
| Black | 26 (26.8) | 9 (42.9) | 13 (25.5) | 4 (16.0) |  |
| Asian | 1 (1.0) |  |  | 1 (4.0) | 0.193 |
| Multiple Race | 7 (7.2) | 1 (4.8) | 2 (3.9) | 4 (16.0) |  |
| Unknown | 1 (1.0) |  | 1 (2.0) |  |  |
| **Ethnicity** | | | | | |
| Hispanic or Latino | 2 (2.1) |  | 2 (3.9) |  | 0.398 |
| Not Hispanic or Latino | 95 (97.9) | 21 (100.0) | 49 (96.1) | 25 (100.0) |  |
| **Length of Stay**, median [Q1, Q3] | 2.0 [1.0, 3.0] | 2.0 [2.0, 4.0] | 1.0 [1.0, 2.0] | 2.0 [1.0, 4.0] |  |
| **Discharge medication** | | | | | |
| Methadone | 2 (2.1) | 1 (4.8) |  | 1 (4.0) | 0.317 |
| Oxycodone | 95 (97.9) | 20 (95.2) | 51 (100.0) | 24 (96.0) |  |
|  | | | | | |
| **Knee Arthroscopy** | 70 | 19 | 26 | 25 |  |
| **Sex** | | | | | |
| Male | 43 (61.4) | 13 (68.4) | 17 (65.4) | 13 (52.0) |  |
| Female | 27 (38.6) | 6 (31.6) | 9 (34.6) | 12 (48.0) | 0.472 |
| **Age**, median [Q1,Q3] | 14.0 [13.0, 15.0] | 14.0 [14.0, 16.0] | 14.0 [13.0, 15.0] | 15.0 [13.0, 15.0] | 0.260 |
| **Race** | | | | | |
| White | 55 (78.6) | 14 (73.7) | 20 (76.9) | 21 (84.0) |  |
| Black | 12 (17.1) | 3 (15.8) | 6 (23.1) | 3 (12.0) |  |
| Asian | 1 (1.4) | 1 (5.3) |  |  | 0.540 |
| Multiple Race | 2 (2.9) | 1 (5.3) |  | 1 (4.0) |  |
| **Ethnicity** | | | | | |
| Hispanic or Latino | 1 (1.4) |  |  | 1 (4.0) | 0.401 |
| Not Hispanic or Latino | 69 (98.6) | 19 (100.0) | 26 (100.0) | 24 (96.0) |  |
| **Length of stay**, median [Q1, Q3] | 0.0 [0.0, 0.0] | 0.0 [0.0, 0.0] | 0.0 [0.0, 0.0] | 0.0 [0.0, 0.0] | 0.704 |
| **Discharge medication** | | | | | |
| Hydrocodone | 26 (37.1) | 3 (15.8) | 9 (34.6) | 14 (56.0) | **0.022** |
| Oxycodone | 44 (62.9) | 16 (84.2) | 17 (65.4) | 11 (44.0) |  |

*p<0.05 considered significant (**bold**)
