# Supplementary material for: Impact of opioid law on prescriptions and satisfaction of pediatric burn and orthopedic patients: An epidemiologic study
Source: PLoS One. 2023 Nov 16;18(11):e0294279. doi: 10.1371/journal.pone.0294279 (PMC10653505; doi:10.1371/journal.pone.0294279)
Supplement: S2 Table — (DOCX) [file pone.0294279.s003.docx]

**S2 Table**: Discharge opioids prescribed pre- and post-law among patients age <11 years and 11+ years

| **Characteristics** | **Pre-law**  **N (%)** | | **Post-law**  **N (%)** | |
| --- | --- | --- | --- | --- |
|  | <11 years | 11+ years | <11 years | 11+ years |
| **BURN INJURY (N)** | 90 | 12 | 67 | 13 |
| **No. of days opioid prescribed at discharge** | | | | |
| Median (IQR) | 7.0 [1.7,10.0] | 1.7 [1.3, 2.9] | 2.3 [1.8, 5.0] | 3.3 [2.0, 4.7] |
| **Total MMEs prescribed at discharge** | | | | |
| Median (IQR) | 3.4 [2.3, 9.0] | 37.5 [25.3, 45.0] | 3.0 [2.3, 8.3] | 15.0 [11.3, 30.0] |
| **Discharge prescription exceeding 30 MME per day** | | | | |
| No (%) | 87 (96.7) | 6 (50.0) | 65 (97.0) | 11 (84.6) |
| Yes (%) | 3 (3.3) | 6 (50.0) | 2 (3.0) | 2 (15.4) |
|  | | | | |
| **KNEE INJURY (N)** | 15 | 35 | 182 | 379 |
| **No. of days opioid prescribed at discharge** | | | | |
| Median (IQR) | 7.5 [4.5, 10.0] | 5.0 [2.5, 5.0] | 5.0 [5.0, 10.0] | 3.8 [2.2, 5.0] |
| **Total MMEs prescribed at discharge** | | | | |
| Median (IQR) | 20.0 [18.6, 25.4] | 20.0 [17.7, 20.0] | 20.0 [20.0, 30.0] | 20.0 [20.0, 20.0] |
| **Discharge prescription exceeding 30 MME per day** | | | | |
| No (%) | 15 (100.0) | 35 (100.0) | 173 (95.1) | 367 (96.8) |
| Yes (%) | 0 (0.0) | 0 (0.0) | 9 (4.9) | 12 (3.2) |
